# Diabetes Mellitus and Increased Tuberculosis Susceptibility: The Role of Short-Chain Fatty Acids

**DOI:** 10.1155/2016/6014631

**Published:** 2015-12-29

**Authors:** Ekta Lachmandas, Corina N. A. M. van den Heuvel, Michelle S. M. A. Damen, Maartje C. P. Cleophas, Mihai G. Netea, Reinout van Crevel

**Affiliations:** Department of Internal Medicine and Radboudumc Center for Infectious Diseases, Radboud University Medical Center, Internal Postal Code 463, P.O. Box 9101, 6500 HB Nijmegen, Netherlands

## Abstract

Type 2 diabetes mellitus confers a threefold increased risk for tuberculosis, but the underlying immunological mechanisms are still largely unknown. Possible mediators of this increased susceptibility are short-chain fatty acids, levels of which have been shown to be altered in individuals with diabetes. We examined the influence of physiological concentrations of butyrate on cytokine responses to *Mycobacterium tuberculosis* (Mtb) in human peripheral blood mononuclear cells (PBMCs). Butyrate decreased Mtb-induced proinflammatory cytokine responses, while it increased production of IL-10. This anti-inflammatory effect was independent of butyrate's well-characterised inhibition of HDAC activity and was not accompanied by changes in Toll-like receptor signalling pathways, the eicosanoid pathway, or cellular metabolism. In contrast blocking IL-10 activity reversed the effects of butyrate on Mtb-induced inflammation. Alteration of the gut microbiota, thereby increasing butyrate concentrations, can reduce insulin resistance and obesity, but further studies are needed to determine how this affects susceptibility to tuberculosis.

## 1. Introduction

Tuberculosis (TB) is the second leading cause of death from an infectious disease worldwide [1]. Susceptibility to TB can be increased by several comorbidities, one of which is type 2 diabetes mellitus (DM) [2]. DM patients present with an overall threefold increased risk of developing active TB [3]. Globally, 15% of TB cases are estimated to be attributable to DM [4] and thus with a predicted increase of DM by 155% over the next 20 years, DM will become an increasingly important factor challenging TB control [5–7].

DM patients exhibit alterations in the immune response against* Mycobacterium tuberculosis* (Mtb), making them more susceptible to infection or progression towards active TB disease and less responsive to treatment [8–11]. However, the underlying biological mechanisms remain largely unknown [12, 13]. DM patients have been associated with dysregulated cytokine responses to Mtb [14–17]. Whilst proinflammatory cytokines are necessary for protection against Mtb, anti-inflammatory cytokines may counteract these effects. Possible factors that may impact the host response in patients with DM are short-chain fatty acids (SCFAs), the main metabolic products of fermentation of nondigestible dietary fibres by the gut microbiota. Numerous reports have demonstrated that DM patients present with an altered composition of their gut microbiota, which subsequently alters their SCFA levels [18–24]. SCFAs strongly modulate immune and inflammatory responses [22, 25–31], thereby influencing the host response to Mtb. SCFAs, of which butyrate (C4) is the most thoroughly studied, act on immune and endothelial cells via at least two mechanisms: activation of G-protein coupled receptors (GPCRs) and inhibition of histone deacetylase (HDAC) [32]. They affect the function of various cell types such as lymphocytes [33, 34], neutrophils [25, 31, 35], and macrophages [28, 36–38]. In light of the emerging role of the microbiota in inflammation and immunity, we hypothesized that SCFAs, and in particular butyrate, may affect the immune response and susceptibility to Mtb in type 2 DM patients.

In this study we investigated the role of physiological concentrations of SCFAs on the cytokine response against Mtb in human peripheral blood mononuclear cells (PBMCs). We subsequently examined a number of possible mechanisms via which altered concentrations of one particular SCFA, C4, might affect the host immune response to Mtb in DM patients. To this purpose, we studied the influence of physiological concentrations of C4 on HDAC activity, immune signalling pathways, the eicosanoid pathway, and cellular metabolism. To our knowledge, this is the first study reporting on the effects of physiological plasma concentrations of C4 on Mtb-induced cellular responses. Physiological plasma concentrations of C4 are in the micromolar range [39], whilst in previous studies C4 has been used in the millimolar range. Thus, this study substantially adds to our knowledge of SCFAs as possible mediators of altered immune responses to Mtb in DM patients.

## 2. Materials and Methods

### 2.1. Human Samples

PBMCs were isolated from buffy coats donated after written informed consent by healthy volunteers to the Sanquin Blood Bank (http://www.sanquin.nl/en/) in Nijmegen. Experiments were conducted according to the principles expressed in the Declaration of Helsinki. Since blood donations were anonymous no tuberculosis skin test or IFN-*γ* release assay was performed. However, the incidence of TB in the Dutch population is extremely low (4/100,000), and Bacillus Calmette-Guérin (BCG) vaccination is not part of the routine vaccination program. Blood donors were not screened for DM as prevalence of DM among people under 45 years of age (median age of blood donors) is about 1.5% and therefore DM is unlikely to be a confounding factor [34].

### 2.2. H37Rv Lysates and Culture

H37Rv Mtb was grown to mid-log phase in Middlebrook 7H9 liquid medium (Difco, Becton Dickinson) supplemented with oleic acid/albumin/dextrose/catalase (OADC) (BBL, Becton Dickinson), washed three times in sterile saline, heat killed, and then disrupted using a bead beater, after which the concentration was measured using a bicinchoninic acid (BCA) assay (Pierce, Thermo Scientific).

### 2.3. Cell Stimulation Experiments

Isolation of PBMCs was performed by differential centrifugation over Ficoll-Paque (GE Healthcare). Cells were adjusted to 5 × 10^6^ cells/mL (Beckman Coulter) and suspended in RPMI 1640 (Gibco) supplemented with 10 *μ*g/mL gentamicin (Lonza), 10 mM L-glutamine (Life Technologies), and 10 mM pyruvate (Life Technologies). 100 *μ*L of PBMCs was incubated in round-bottom 96-well plates (Greiner), pretreated with SCFAs for 1 h, and stimulated with 1 *μ*g/mL of H37Rv lysate or 10 ng/mL LPS (Sigma-Aldrich,* E. coli* serotype 055:B5). Cells were incubated for 24 h or 7 days at 37°C in a 5% CO_2_ environment (*n* = 6 to 11). Alternatively, PBMCs were pretreated for 1 hour (37°C, 5% CO_2_) with ranolazine (ITK Diagnostics), trimetazidine (Sigma), pertussis toxin (Enzo Life Sciences), etomoxir (Sigma) (inhibitors of *β*-oxidation, *n* = 3), aspirin (Aspégic injection powder, *n* = 3), cycloheximide (Sigma, *n* = 6 to 7), anti-IL-10 antibody IgG2a (BioLegend, *n* = 10 to 12), or IgG2a isotype control (BioLegend, *n* = 10 to 12) prior to stimulation. Cell culture supernatants were collected and stored at −20°C for cytokine measurements, performed by ELISA: TNF-*α*, IL-1*β*, IL-17A, IL-22, and IL-1Ra (R&D Systems) and IL-6, IFN-*γ*, and IL-10 (Sanquin).

### 2.4. Quantification of Gene Expression

For quantitative real-time PCR (qPCR) analysis RNA was isolated from PBMCs using TRIzol reagent (Invitrogen Life Technologies) according to the manufacturer's protocol. RNA was transcribed into complementary DNA (cDNA) by reverse transcription using iScript cDNA synthesis kit (Bio-Rad, Hercules, CA). Primer sequences (Biolegio) are given in Table 1. Power SYBR Green PCR Master Mix (Applied Biosystems) was used for qPCR on an AB StepOnePlus real-time PCR system (Applied Biosystems). qPCR data were normalized to the housekeeping gene human *β*2M (*n* = 3 to 10).

### 2.5. Protein Phosphorylation Measurements

Western blotting was carried out using a Trans-Blot Turbo system (Bio-Rad) according to manufacturer's instructions. 5 × 10^6^ PBMCs were lysed in 100 *μ*L lysis buffer. The resulting lysate was used for Western blot analysis. Equal amounts of protein were separated by SDS-PAGE on 4–15% polyacrylamide gels (Bio-Rad) and transferred to PVDF (Bio-Rad) membranes. Membranes were blocked for 1 h and then incubated overnight with primary antibody (dilution 1 : 1000) in 5% (w/v) BSA or milk in TBS-Tween buffer (TBS-T). Blots were washed in TBS-T 3 times and incubated with HRP-conjugated anti-rabbit antibody (1 : 5000; Sigma) in 5% (w/v) milk in TBS-T for 1 h at room temperature (RT). After washing, blots were developed with ECL (Bio-Rad) following manufacturer's instructions. Primary antibodies used were rabbit anti-p38 MAPK, rabbit anti-phospho-p38 MAPK, rabbit anti-ERK1/2 (p44/p42 MAPK), rabbit anti-phospho-ERK1/2 (P44/42 MAPK, T202/Y204), and rabbit anti-phospho-JNK (T183/Y185) (all Cell Signalling) (*n* = 2).

### 2.6. Metabolite Measurements

Lactate was measured from cell culture supernatants using a coupled enzymatic assay in which lactate was oxidised and the resulting H_2_O_2_ was coupled to the conversion of Amplex Red reagent to fluorescent resorufin by HRP (horseradish peroxidase). 30 *μ*L of lactate standard or 200x diluted sample was added to 30 *μ*L of reaction mix. The 30 *μ*L of reaction mix consisted of 0.6 *μ*L of 10 U/mL HRP (Sigma), 0.6 *μ*L of 100 U/mL lactate oxidase (Sigma), 0.3 *μ*L of 10 mM Amplex Red reagent (Life Technologies), and 28.5 *μ*L PBS. Samples were incubated for 20 min at RT and fluorescence (excitation/emission maxima = 570/585 nm) was measured on an ELISA reader (BioTek) (*n* = 3 to 5).

Measurements of the NAD^+^/NADH redox ratio were adapted from Zhu and Rand [40]. Briefly, 1.5 million stimulated PBMCs were lysed in 75 *μ*L of homogenization buffer (10 mM nicotinamide (Sigma), 10 mM Tris-Cl (Sigma), and 0.05% (w/v) Triton X-100 (Sigma), pH 7.4). The lysate was centrifuged at 12000 g for 1 min. From the resulting supernatants two 18 *μ*L aliquots were removed and either 2 *μ*L of 0.2 M HCl or 0.2 M NaOH was added to each aliquot. The samples were heated for 30 min at 65°C and after incubation 2 *μ*L of opposite reagent (NaOH or HCl) was added to each aliquot. 5 *μ*L of sample or NAD^+^ (*β*-nicotinamide adenine dinucleotide hydrate; Sigma) standard was then mixed with 85 *μ*L of reaction mix and 60 *μ*L of fluorescence mix in a black 96-well plate. The reaction mix consisted of 100 mM bicine (N,N-bis(2-hydroxyethyl)glycine; Sigma), 0.6 mM ethanol (Sigma), and 5 mM EDTA (Life Technologies). The fluorescence mix consisted of 0.5 mM PMS (phenazine methosulfate; Sigma), 0.05 mM resazurin (Sigma), and 0.2 mg of ADH (alcohol dehydrogenase; Sigma). The reaction was incubated for 15 min at RT and fluorescence (excitation/emission maxima = 540/586 nm) was measured on an ELISA reader (BioTek) (*n* = 3 to 5).

### 2.7. HDAC Activity Assay

HDAC Fluorometric Cellular Activity Assay BML-AK503 (FLUOR DE LYS, Enzo Life Sciences, Inc., Farmingdale, NY) was used to determine HDAC activity in PBMCs pretreated with C4 (30 min) and then stimulated with H37Rv (30 min). Subsequently PBMCs were incubated with acetylated substrate for 2 hours, after which a developer was added to generate a fluorescent signal from the deacetylated substrate. Fluorescence was measured on a microplate reader (BioTek). Trichostatin A (TSA) was used as a positive control for HDAC inhibition (*n* = 5 to 6).

### 2.8. Flow Cytometry

PBMCs were treated with 50 *μ*mol C4 for 1 h and stimulated with 1 *μ*g/mL H37Rv or 10 ng/mL LPS for 7 days. Subsequently cells were restimulated with 200 *μ*L RPMI supplemented with 10% serum, Golgi-plug inhibitor (GPI Brefeldin A; 1 *μ*g/mL, BD Pharmingen), PMA (phorbol 12-myristate 13-acetate; 50 *μ*g/mL, Sigma-Aldrich), and ionomycin (1 *μ*g/mL, Sigma-Aldrich) for 4–6 h at 37°C and 5% CO_2_. Cells were then washed with PBA (PBS 1% BSA (albumin from bovine serum)) and stained extracellularly for 30 min with CD4-PeCys7 (ITK) for T-helper 17 (Th17) cells at 4°C. Next, cells were washed and permeabilized by fix and perm buffer (eBioscience) according to the manufacturer's protocol for 45–60 min at 4°C. Finally cells were washed and resuspended in 300 *μ*L PBA to be measured using the Cytomics FC500 (Beckman Coulter) (*n* = 8).

Cell death was measured by staining PBMCs with Annexin V-FITC (BioVision) and Propidium Iodide (PI) (Invitrogen Molecular Probes). Cells were incubated in the dark on ice with Annexin-V staining solution (RPMI supplemented with 5 mM CaCl_2_ and 0.1 *μ*L/mL Annexin-V) for 15 minutes. Subsequently PBMCs were stained with PI for 5 minutes. Cells were measured with the Cytomics FC500 (Beckman Coulter, Woerden, Netherlands), and data were analysed using CXP analysis software v2.2 (Beckman Coulter) (*n* = 3 to 5).

### 2.9. Statistical Analysis

All data were analysed using a paired nonparametric Wilcoxon signed-rank test, as the data were not normally distributed. Differences were considered statistically significant at *p* value < 0.05. Data are shown as cumulative results of levels obtained in all volunteers (means ± SEM).

## 3. Results

### 3.1. Short-Chain Fatty Acids Inhibit Mtb-Induced Cytokine Responses

**Figure 1:**
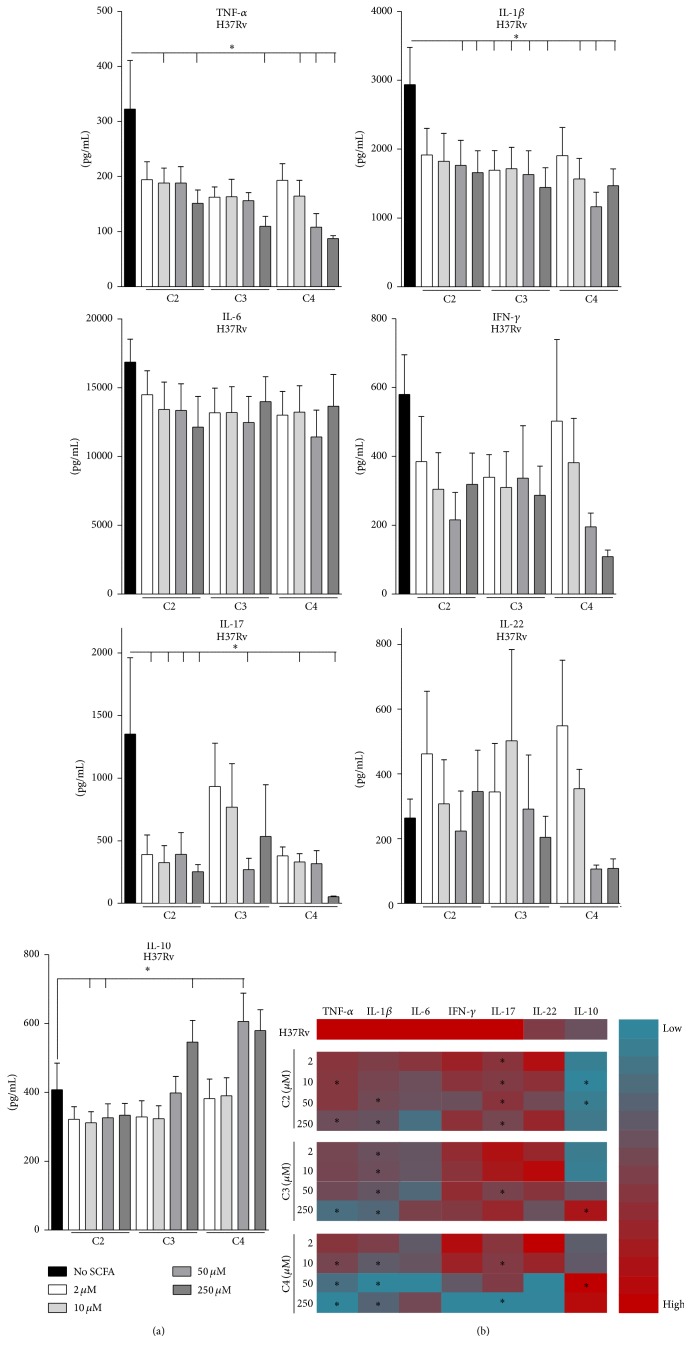
Short-chain fatty acids inhibit Mtb-induced cytokine responses. (a) PBMCs were preincubated with 2–250 *μ*M SCFAs for 1 h prior to stimulation with Mtb lysate for 24 h and 7 d. Hereafter TNF-*α*, IL-6, IL-10, IFN-*γ*, IL-17, and IL-22 were measured in supernatants by ELISA. Data are means ± SEM (*n* = 6), using Wilcoxon signed-rank test, representative of 2 independent experiments. ^*∗*^ *p* < 0.05. (b) Heat map of log-transformed mean cytokine responses as measured by ELISA, showing cytokines upregulated (red) and downregulated (blue) upon H37Rv stimulation in the presence of different doses of SCFAs. Cytokine responses are shown as compared to H37Rv stimulation alone. ^*∗*^ *p* < 0.05.

DM is associated with altered gut microbiota and consequently altered SCFA levels [18–22]. In line with current literature [22, 25–31], we hypothesized that SCFAs have the potential to influence the host inflammatory response against Mtb. In particular we investigated the effects of varying doses of acetate (C2), propionate (C3), and butyrate (C4) on H37Rv-induced cytokine responses, with RPMI as negative control and LPS as positive control (Figure 1). SCFAs themselves did not induce cytokine production (results not shown) but significantly affected H37Rv-induced cytokine release. C2, C3, and C4 significantly, dose-dependently decreased H37Rv-induced production of proinflammatory cytokines TNF-*α*, IL-1*β*, and IL-17, while nonsignificant effects were found for IL-6, IFN-*γ*, and IL-22 production. In contrast, C3 and C4 induced a significant increase in H37Rv-induced production of the anti-inflammatory cytokine IL-10. Similarly, C3 and C4 but not C2 decreased LPS-induced production of TNF-*α* and IL-6, while the release of IL-1*β* was significantly decreased in response to all three SCFAs (results not shown). LPS did not induce production of IFN-*γ*, IL-17, or IL-22. Moreover, all three SCFAs incurred a dose-dependent, nonsignificant decrease in LPS-induced IL-10 production (results not shown).

Overall, C4 resulted in some of the most significant changes in cytokine responses (Figure 1(b)). Moreover, the potency of butyrate in reducing cytokine responses to H37Rv and LPS was greater than that for the other SCFAs. Importantly, changes in cytokine levels could not be explained by altered pH levels or cell death (Supplementary Figure 1 A and B in Supplementary Material available online at http://dx.doi.org/10.1155/2016/6014631). Therefore, following this screen, we continued our study with C4 at a concentration of 50 *μ*M, which is physiologically relevant because it is comparable to human plasma concentrations [39].

### 3.2. Influence of Butyrate on HDAC Expression and Activity

**Figure 2:**
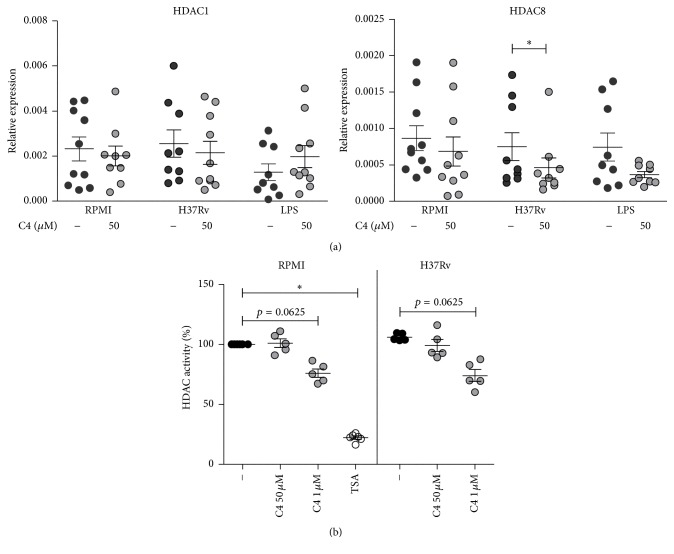
Influence of butyrate on HDAC expression and activity. (a) PBMCs were preincubated with 50 *μ*M C4 for 1 h prior to stimulation with Mtb lysate or LPS for 4 h. Gene expression levels of HDAC1 and HDAC8 were measured by qPCR. Data are means ± SEM (*n* = 10), using Wilcoxon signed-rank test, representative of 3 independent experiments. ^*∗*^ *p* < 0.05. (b) Percentage of general HDAC activity relative to RPMI stimulated PBMCs, as measured by levels of substrate deacetylation after 30 min of preincubation with C4 (50 *μ*M) and 30 min of stimulation with Mtb lysate. Data are means ± SEM (*n* = 5 to 6), using Wilcoxon signed-rank test, representative of 3 independent experiments. ^*∗*^ *p* < 0.05.

Butyrate is reported to be a strong HDAC inhibitor. Since this might account for its anti-inflammatory effects [41–44], we examined the effect of C4 on HDAC expression and activity. C4 significantly decreased HDAC8 but not HDAC1 gene expression upon H37Rv stimulation of PBMCs (Figure 2(a)). Consistent with previous reports [36, 42–44], C4 at a high dose of 1 mM decreased HDAC activity upon both RPMI and H37Rv stimulation. However, different from its effect on gene expression, C4 at a physiological dose of 50 *μ*M had no effect on actual HDAC activity (Figure 2(b)), while trichostatin A (TSA, positive control) strongly decreased HDAC activity. These data suggest that butyrate's inhibition of HDAC activity is unlikely to play a role in the effects of low doses of C4 on Mtb-induced inflammatory responses and stresses the importance of studying the effects of butyrate at physiologically relevant concentrations.

### 3.3. The Effects of Butyrate on TLR-Signalling Mediators and the Eicosanoid Pathway

**Figure 3:**
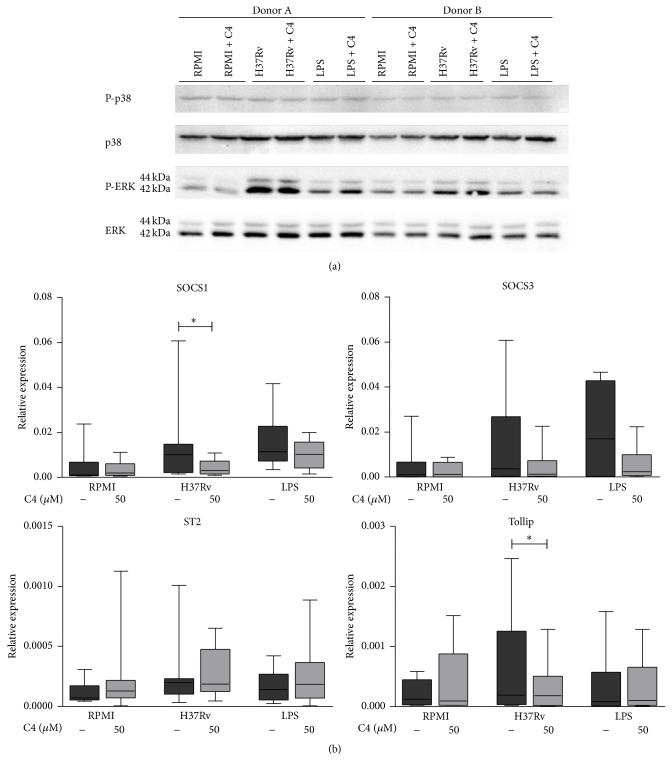
The effects of butyrate on TLR signalling mediators. (a) PBMCs were preincubated with 50 *μ*M C4 (1 h) and stimulated with Mtb lysate or LPS. Cell lysates were harvested at 30 min after stimulation. Phospho-p38, p38, phospho-ERK, and ERK protein levels were determined by Western blot using specific antibodies (*n* = 2). (b) Gene expression levels of SOCS1, SOCS3, ST2, and Tollip in PBMCs preincubated with 50 *μ*M C4 (1 h) and stimulated with Mtb lysate or LPS (4 h) as measured by qPCR. The box plot represents median with first and third quartiles; the whiskers represent minimum and maximum values. *n* = 10, using Wilcoxon signed-rank test, representative of 3 independent experiments. ^*∗*^ *p* < 0.05.

Signalling of Toll-like receptors (TLRs), important receptors for Mtb recognition [45–47], is controlled by feedback mechanisms regulated by several intracellular kinases [48, 49]. Because impaired Mtb recognition and insufficient TLR signalling may account for the anti-inflammatory effects of C4, we examined whether C4 affected these feedback loops. However, C4 had no effect on phosphorylation of the MAP kinases p38, ERK (Figure 3(a)), or JNK (Supplementary Figure 2). C4 has also been reported to induce expression of inhibitors of TLR signalling pathways [50], but we found that C4 significantly decreased mRNA expression of TLR signalling inhibitors SOCS1 and Tollip and did not affect expression of SOCS3 or ST2 (Figure 3(b)). Of note, these results were not explained by cell death (Supplementary Figure 1 B).

Aside from TLR signalling, C4 possibly exerts its anti-inflammatory effects through modulation of the eicosanoid pathway. Eicosanoids, oxygenated metabolites of arachidonic acid, modulate the host immune response to Mtb [51–55]. C4 has been reported to upregulate key enzymes of the eicosanoid pathway upon LPS stimulation [30], but a reverse effect has also been described [56]. We did not observe a significant impact of C4 on transcript levels of cyclooxygenase 2 (COX-2), one of the main eicosanoid enzymes, upon H37Rv or LPS stimulation (Supplementary Figure 3 A). Alternatively, C4 has been described to induce release of the anti-inflammatory prostaglandin PGE_2_ [26, 30, 57]. Inhibition of PGE_2_ with aspirin could not counteract the inhibitory effects of C4 on TNF-*α* and IL-1*β* cytokine responses upon either H37Rv or LPS stimulation (Supplementary Figure 3 B). The eicosanoid pathway is therefore unlikely to be the mediator pathway through which C4 exerts its anti-inflammatory effects.

### 3.4. Influence of Butyrate on Cellular Metabolism

**Figure 4:**
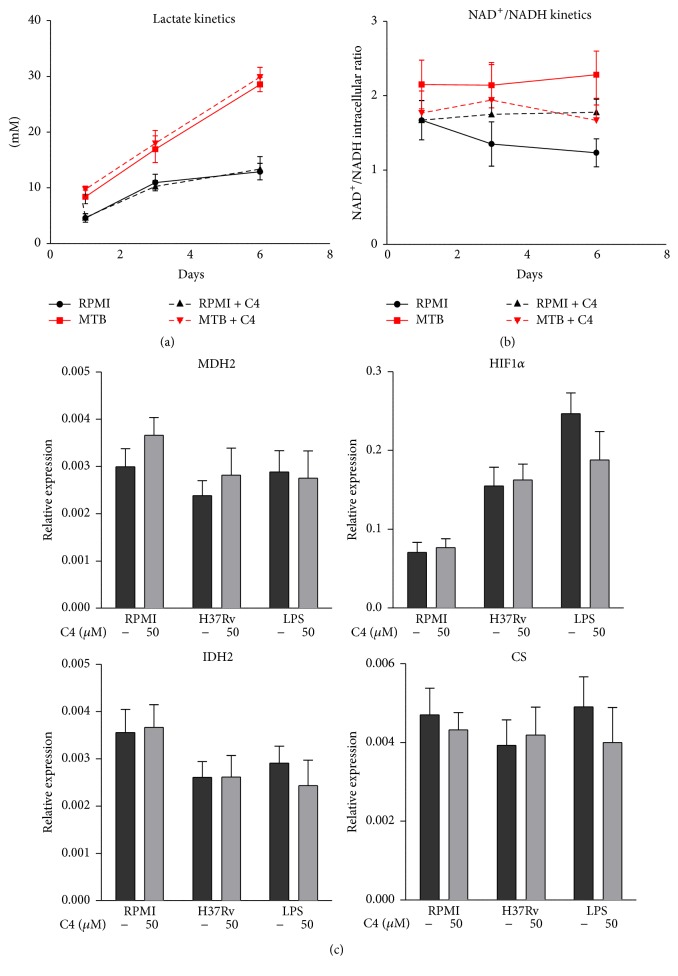
Influence of butyrate on cellular metabolism. (a and b) Kinetics of lactate production (a) and intracellular NAD^+^/NADH ratios (b) from days 1, 3, and 7 of PBMCs preincubated with 50 *μ*M C4 (1 h) with and without stimulation with Mtb lysate. Data are means ± SEM (*n* = 3 to 5), using Wilcoxon signed-rank test, representative of 1-2 independent experiments. (c) Expression levels of glycolysis and TCA cycle genes in PBMCs preincubated with 50 *μ*M C4 (1 h) and stimulated with Mtb lysate or LPS (4 h) as measured by qPCR. Data are means ± SEM (*n* = 6), using Wilcoxon signed-rank test, representative of 2 independent experiments.

Another possible explanation for butyrate's anti-inflammatory effects is its influence on cellular metabolism. A recent paper described that microbiota have a strong effect on energy homeostasis in the mammalian colon and showed that C4 regulates different aspects of energy metabolism acting as an important energy source for colonocytes [58]. Contrary to this previous study, we observed no effects of C4 on cellular lactate production, the NAD^+^/NADH redox ratio, TCA cycle gene expression (Figure 4), or *β*-oxidation (Supplementary Figure 4). These data strongly suggest that C4 modulates the immune response to Mtb independently of cellular metabolism.

### 3.5. Butyrate Transcriptionally Influences Cytokine Responses to Mtb, Possibly Mediated through IL-10 Induction

**Figure 5:**
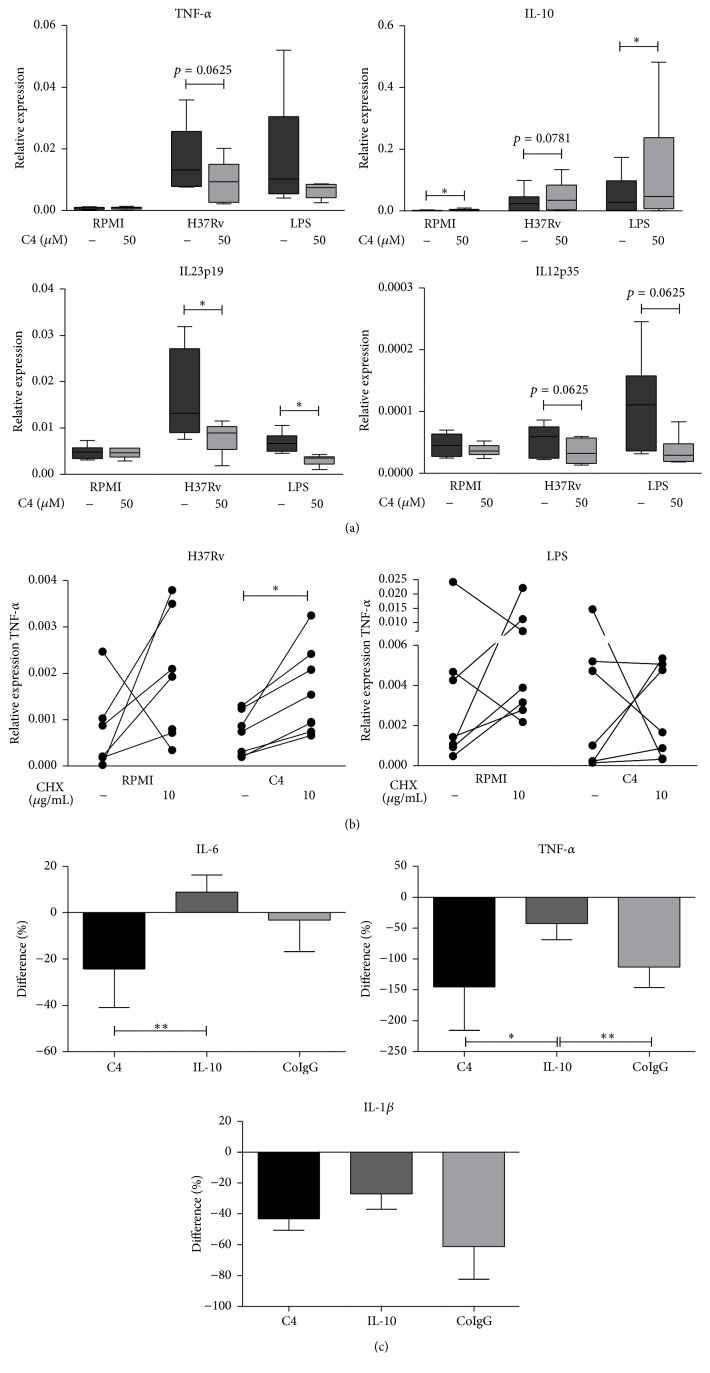
Butyrate transcriptionally influences cytokine responses to Mtb, possibly mediated through IL-10. (a) Cytokine gene expression levels in PBMCs preincubated with 50 *μ*M C4 for 1 h prior to stimulation with Mtb lysate or LPS for 4 h, as measured by qPCR. The box plot represents median with first and third quartiles; the whiskers represent minimum and maximum values. *n* = 6 to 10, using Wilcoxon signed-rank test, representative of 2+ independent experiments. ^*∗*^ *p* < 0.05. (b) To block translation, PBMCs were preincubated with cycloheximide (CHX) for 1 h prior to 1 h incubation with C4 (50 *μ*M). TNF-*α* transcript levels were measured by qPCR 4 h after stimulation with Mtb lysate or LPS. Data are single values (*n* = 6 to 7), using Wilcoxon signed-rank test, representative of 3 independent experiments. ^*∗*^ *p* < 0.05. (c) To block IL-10 activity, PBMCs were preincubated with IL-10 and C4 (50 *μ*M) for 1 h. IL-6, TNF-*α*, and IL-1*β* production was measured by ELISA after 24 h of stimulation with Mtb lysate. Data are means ± SEM (*n* = 10 to 12), using Wilcoxon signed-rank test, representative of 4 independent experiments.

We next examined whether the inhibitory effect of C4 on Mtb-induced proinflammatory cytokine responses, with a concomitant increase in anti-inflammatory IL-10 production (Figure 1) and decrease in Th17 proliferation (Supplementary Figure 5 A), was also present at the level of gene transcription. C4 led to a decrease in TNF-*α*, IL-12, and IL-23 mRNA levels upon H37Rv stimulation and a parallel increase in IL-10 mRNA (Figure 5(a)), while no effect on production of the anti-inflammatory cytokine IL-1Ra was observed (Supplementary Figure 5 B). These data point to IL-10 as a possible intermediary mediator of the anti-inflammatory effects of C4. We therefore assessed whether removing IL-10 protein from the cellular environment could counteract the inhibitory effects of C4. To this end, we pretreated PBMCs with cycloheximide (CHX), an inhibitor of translation. Stimulation of PBMCs with H37Rv in the presence of C4 in combination with CHX resulted in higher TNF-*α* responses, as compared to incubation with H37Rv and C4 alone. Upon LPS stimulation, this effect was not present (Figure 5(b)). We subsequently examined whether blocking IL-10 specifically using an anti-IL-10 antibody could counteract the inhibitory effects of C4 on proinflammatory cytokine response. Blocking IL-10 completely restored IL-6 cytokine responses in response to H37Rv and C4, while TNF-*α* and IL-1*β* production was partly restored (Figure 5(c)). This suggests an important role for intermediary protein synthesis, specifically IL-10, in mediating the anti-inflammatory effects of C4.

## 4. Discussion

DM is associated with a threefold increased risk of active TB, but the underlying immunological mechanisms remain largely unknown [3, 12, 13]. Alterations in the gut microbiota of DM patients are associated with changes in plasma SCFA concentrations. Multiple papers have reported a decrease in C4-producing bacteria in type 2 DM patients [18, 19, 21, 23, 24]. We here show that SCFAs, especially C4, exhibit anti-inflammatory properties; low doses of C4 decreased Mtb-induced proinflammatory cytokine responses on both the transcriptional level and the translational level, while production of IL-10 was increased. This anti-inflammatory effect was independent of HDAC activity, Toll-like receptor signalling, the eicosanoid pathway, or cellular metabolism.

We observed a general anti-inflammatory effect of C2, C3, and C4 on Mtb-induced cytokine production. C4 induced some of the most significant and most potent changes in cytokine responses, which is in line with published results [29], although our study is the first to examine the effects of physiological concentrations of SCFAs on Mtb-induced cytokine responses* in vitro*. Several observations were made regarding the effect of SCFA on cytokines. Firstly, the inhibitory effect of all three SCFAs on production of TNF-*α* and IL-1*β* was comparable for Mtb and LPS stimulation. However, while C3 and C4 had a clear effect on LPS-induced IL-6 release, this was not found for Mtb. This suggests that SCFAs do not affect Mtb-induced IL-6, although IL-6 has been assigned an important role in Mtb host responses [59–62]. Secondly, C2, C3, and C4 had a much stronger inhibitory effect on T-cell derived cytokine IL-17 than on T-cell derived cytokines IFN-*γ* and IL-22. Because C4 also strongly decreased Th17 proliferation (Supplementary Figure 5 A), SCFAs may affect Th17 subsets more than other T-cell subsets. This may be of great relevance since Th17 cells, and IL-17 in particular, have been reported to be essential in protective immunity against Mtb [63, 64] but inversely associated with DM complications [65–67]. Lastly, the stimulatory effect of C3 and C4 on anti-inflammatory IL-10 release was Mtb-specific and was not seen with LPS stimulation. IL-10 has been delineated as an important mediator in Mtb infection: it has been reported to block bacterial killing in Mtb-infected macrophages, suppress multinucleated giant cell formation and cytokine production, and inhibit the development of protective immunity [68–74]. In contrast to TB, IL-10 may have a protective role in type 2 DM by reducing insulin resistance and obesity [75–77]. Therefore, the increase in IL-10 production we see as induced by C4 is very relevant for the course of both DM and TB disease.

We examined several possible mechanisms underlying the effect of C4 on cytokine production, starting with HDAC activity, which is known to be inhibited by SCFAs. C4 at a physiological low dose of 50 *μ*M had little effect, while millimolar concentrations of C4 (as used in other studies [36, 41–44]) decreased HDAC activity upon H37Rv stimulation. This is expected as IC_50_ values of HDAC inhibition by C4 are >100 *μ*M, depending on the class of HDAC [43]. The strongest effect was noted for HDAC8, which is reported to be most sensitive to C4 [43]. This argues that physiological C4 concentrations in human plasma do not exert HDAC inhibition and underlines the importance of using physiological concentrations within* in vitro* experimental models.

In contrast to a previous study [50], we observed a decreased gene expression of the TLR modulatory factors SOCS1 and Tollip when PBMCs were stimulated in the presence of C4, which thus cannot explain the inhibitory effects on cytokine production. This, together with our data showing that C4 does not affect MAP kinase activity, suggests that C4 does not act at the level of TLR signalling, as shown previously [36].

As a third possible mechanism, we assessed whether C4 exerts its effects through eicosanoid metabolism. The eicosanoid pathway is under influence of SCFAs [30, 56] and may modulate the host response to Mtb [51–55]. C4 did not affect expression of COX-2, a key enzyme in the eicosanoid pathway, in contrast to previous reports that used supraphysiological C4 concentrations [30, 56]. In addition, inhibition of the eicosanoid pathway using aspirin did not counteract the effects of C4. Therefore, the eicosanoid pathway is unlikely to be involved in mediating the effects of C4.

The effect of diabetes on the host immune response to Mtb might also be explained by altered cellular metabolism, with a possible role for SCFA. Cellular metabolism is increasingly linked to immunology [78–80]. One previous study noted that C4 influences metabolic processes in colonocytes [58], which use butyrate as their primary energy source [58]. However, we did not observe any effect of C4 on lactate production, the redox status, TCA cycle gene expression, or *β*-oxidation in PBMCs. We therefore conclude that cellular metabolism does not mediate the effect of C4 on Mtb-induced cytokine production.

Finally, we further examined the effect of C4 on the anti-inflammatory cytokine IL-10. IL-10 is detrimental to TB outcome, while it may improve DM symptoms [68–77]. In line with previous studies [33, 81, 82], we report an upregulation in IL-10 production induced by C4. Removal of all intermediary protein, including IL-10, from PBMCs stimulated with H37Rv and C4 led to a significant increase in TNF-*α* transcript, thereby counteracting the decrease in TNF-*α* production induced by C4. Moreover, blocking IL-10 specifically fully restored IL-6 responses in PBMCs stimulated with H37Rv and C4 and partly restored TNF-*α* and IL-1*β* responses. These data suggest that the anti-inflammatory cytokine IL-10 may play a role in the inhibitory effects of C4 on Mtb-induced inflammatory responses.

Currently, much research focuses on modulation of the gut microbiota in order to treat obesity and type 2 DM [83–86]. Administration of sodium butyrate or butyrate-inducing probiotics in mice significantly increased plasma insulin levels and insulin sensitivity and suppressed body weight gain [87–89]. The anti-inflammatory effects of C4 may attenuate the chronic inflammatory state associated with type 2 DM, thereby improving DM symptoms. If chronic inflammation is a causal factor of the impaired host response to Mtb in type 2 DM patients, attenuation of this hyperinflammatory state may improve not only DM but also TB outcome in patients with coincident DM and TB disease.

Some limitations of our study need to be addressed. Firstly, we studied the effects of C4 on Mtb-induced inflammation in PBMCs* in vitro*. SCFA levels have been shown to be altered in DM patients [18–22], but this* in vitro* model does not include other aspects of the pathophysiology of DM such as hyperglycemia, hyperinsulinemia, or dyslipidemia, phenomena which have also been reported to affect immunity [90–94]. Furthermore, DM medications possibly interfere with the intestinal microbiota and immune responses in patients [95–97]. It is therefore unclear how accurately our* in vitro* model reflects the* in vivo* situation in DM patients.

In conclusion, we show an anti-inflammatory effect of low, physiological doses of C4 on Mtb-induced inflammatory responses. The anti-inflammatory cytokine IL-10 may play a role in mediating the inhibitory effects of C4 on the host immune response to Mtb. Further studies are needed to precisely explore the pathways by which physiological concentrations of C4 exert their anti-inflammatory effects and to define the mechanism of increased TB sensitivity in type 2 DM patients. Moreover, current research on modulating gut microbiota in DM should include its possible effects on TB.

## Supplementary Material

The efficiency of the subcutaneous administration of GK-1 along with the immunotherapy based in bone marrow dendritic cells loaded with MAGE-AX was proven when this treatment induced in mice with melanoma increased survival and diminished tumor- diameter until they disappeared, a phenomenon known as tumor regression.

## Figures and Tables

**Table 1:** Primer sequences used for gene expression measurements by qPCR.

Target	Forward 5′ → 3′	Reverse 5′ → 3′
h-*β*2M	ATGAGTATGCCTGCCGTGTG	CCAAATGCGGCATCTTCAAAC
h-COX-2	CTGGCGCTCAGCCATACAG	CGCACTTATACTGGTCAAATCCC
h-CS	GGTGGCATGAGAGGCATGAA	TAGCCTTGGGTAGCAGTTTCT
h-HDAC1	CCGCATGACTCATAATTTGCTG	ATTGGCTTTGTGAGGGCGATA
h-HDAC8	TCGCTGGTCCCGGTTTATATC	TACTGGCCCGTTTGGGGAT
h-HIF1-*α*	GAACGTCGAAAAGAAAAGTCTCG	CCTTATCAAGATGCGAACTCACA
h-IDH2	CGCCACTATGCCGACAAAAG	ACTGCCAGATAATACGGGTCA
h-IL-10	CAACCTGCCTAACATGCTTCG	TCATCTCAGACAAGGCTTGGC
h-IL-1*β*	GCCCTAAACAGATGAAGTGCTC	GAACCAGCATCTTCCTCAG
h-IL12p35	CCTTGCACTTCTGAAGAGATTGA	ACAGGGCCATCATAAAAGAGGT
h-IL23p19	CTCAGGGACAACAGTCAGTTC	ACAGGGCTATCAGGGAGC
h-MDH2	TCGGCCCAGAACAATGCTAAA	GCGGCTTTGGTCTCGATGT
h-SOCS1	TTTTCGCCCTTAGCGTGAAGA	GAGGCAGTCGAAGCTCTCG
h-SOCS3	TGCGCCTCAAGACCTTCAG	GAGCTGTCGCGGATCAGAAA
h-ST2	TTGTCTACCCACGGAACTACA	GCTCTTTCGTATGTTGGTTTCCA
h-TNF-*α*	CCTCTCTCTAATCAGCCCTCTG	GAGGACCTGGGAGTAGATGAG
h-Tollip	TGGGCCGACTGAACATCAC	GTGGATGACCTTATTCCAGCG
