# Supplementary material for: Diabetes Mellitus and Increased Tuberculosis Susceptibility: The Role of Short-Chain Fatty Acids
Source: J Diabetes Res. 2015 Dec 29;2016:6014631. doi: 10.1155/2016/6014631 (PMC4709651; doi:10.1155/2016/6014631)

Supplementary Figure 1

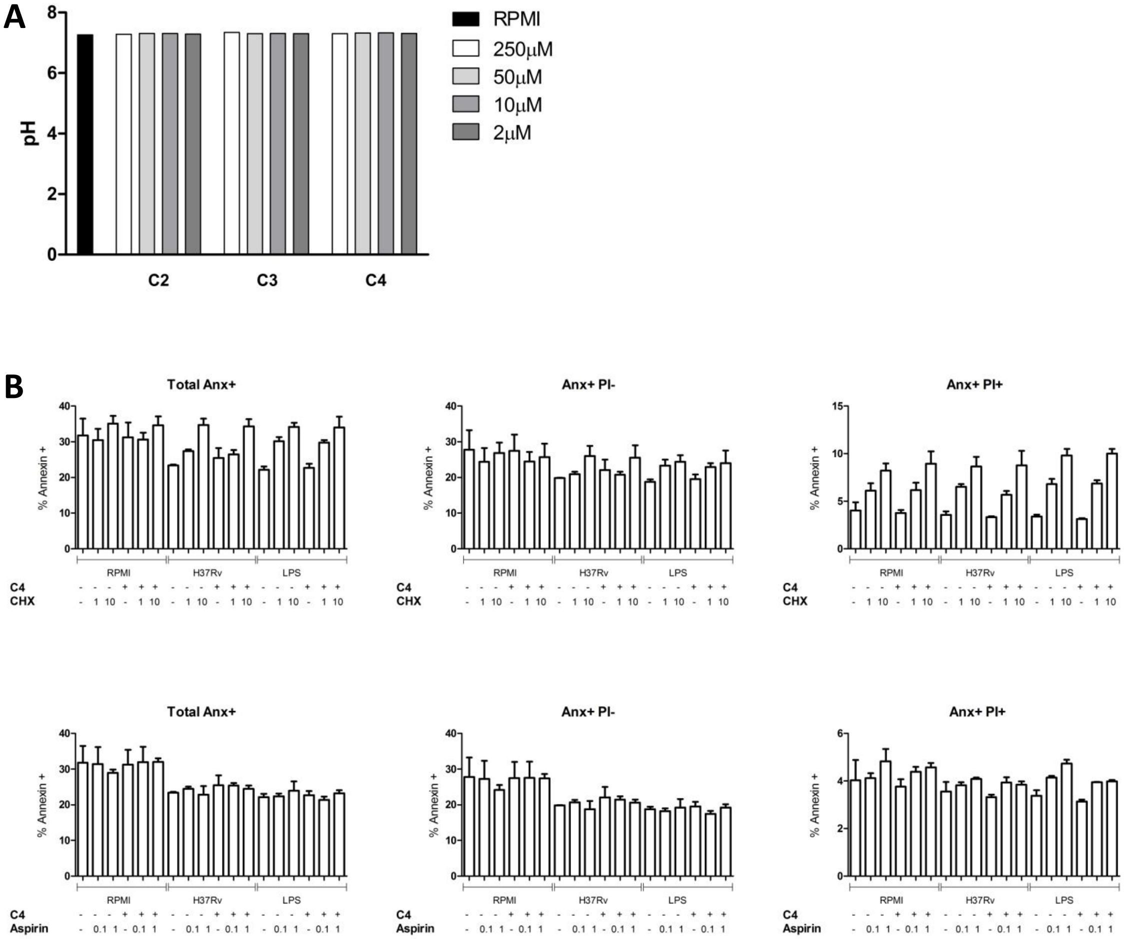

Supplementary Figure 2

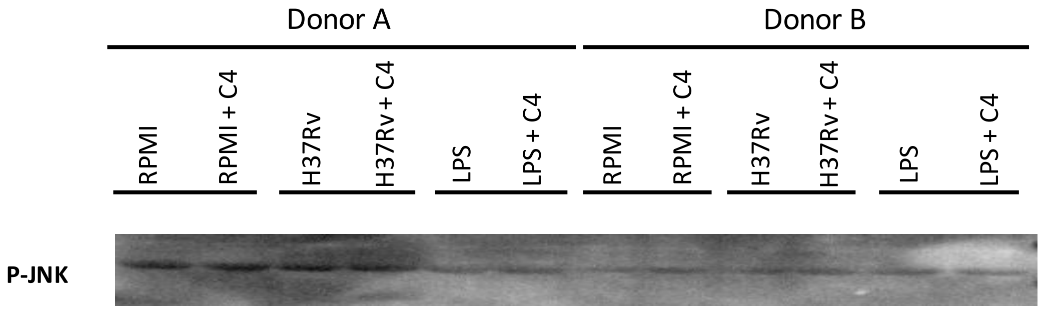

Supplementary Figure 3

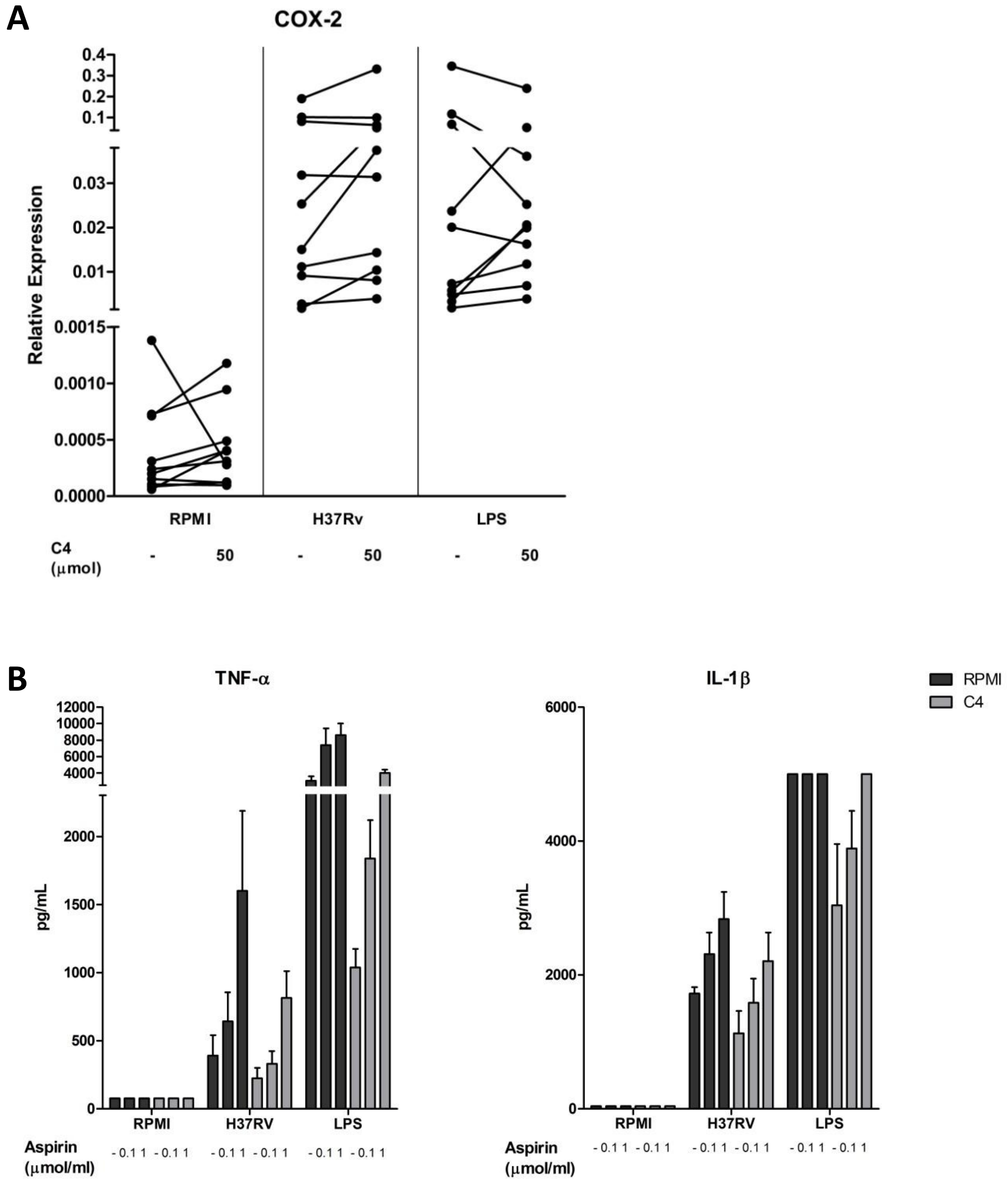

Supplementary Figure 4

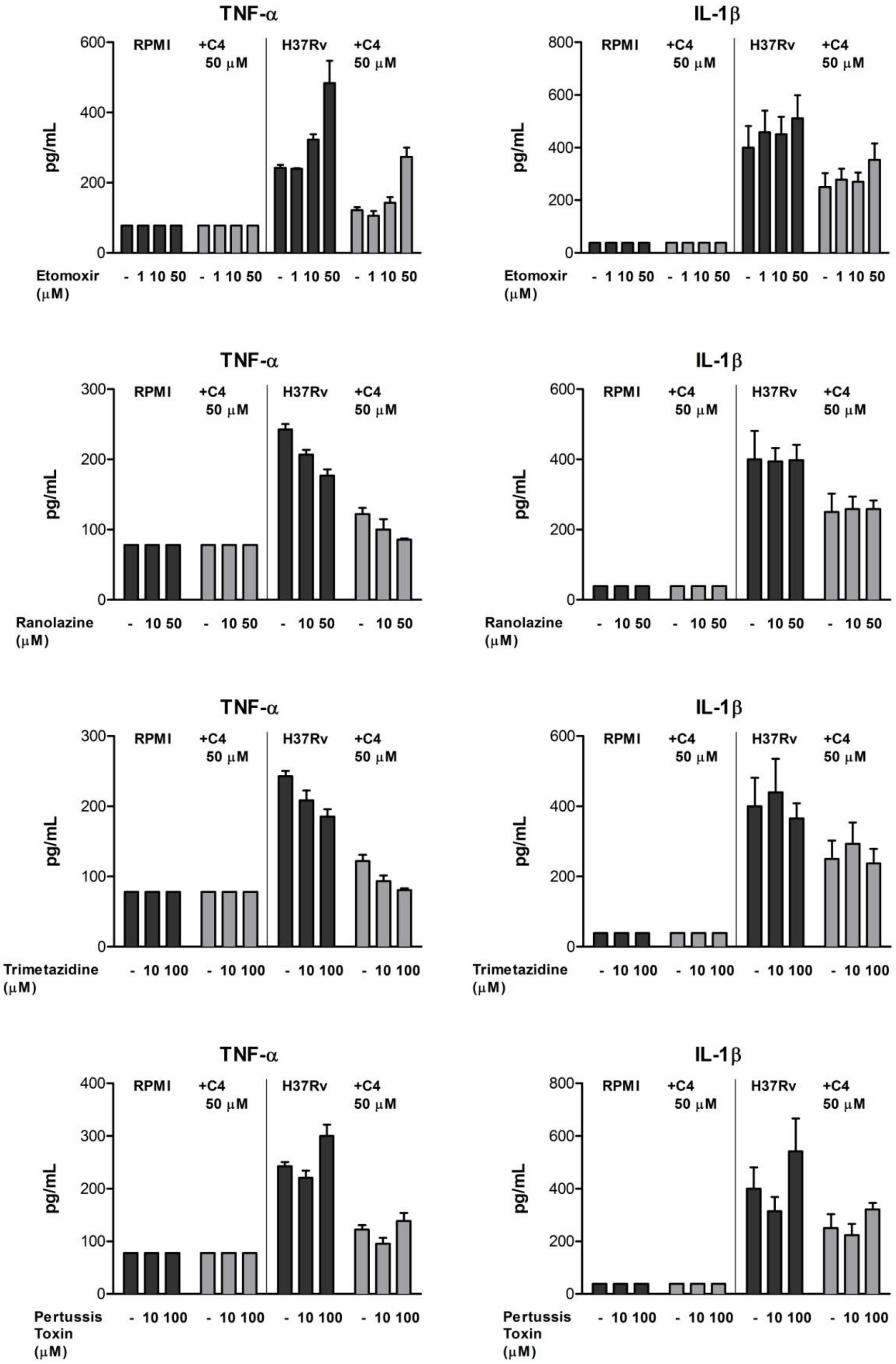

Supplementary Figure 5

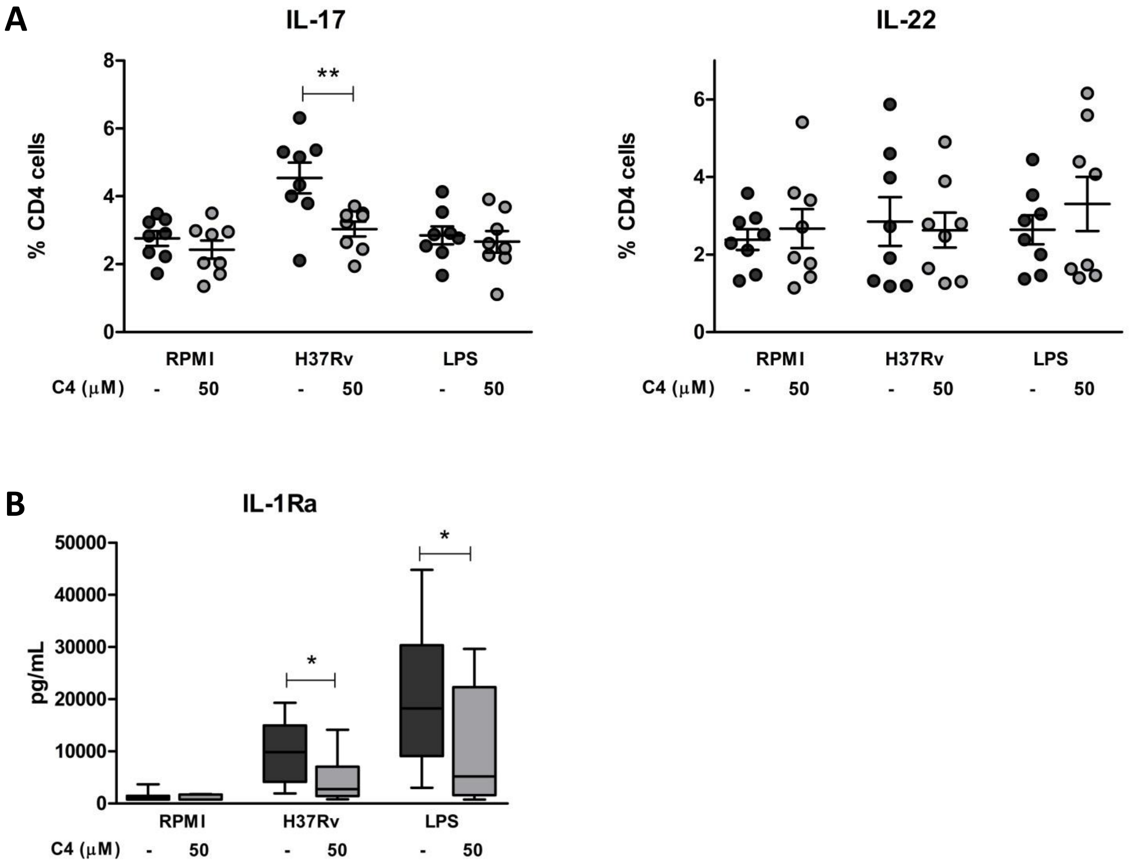

Supplement: Supplementary file 1 — The efficiency of the subcutaneous administration of GK-1 along with the immunotherapy based in bone marrow dendritic cells loaded with MAGE-AX was proven when this treatment induced in mice with melanoma increased survival and diminished tumor- diameter until they disappeared, a phenomenon known as tumor regression. [file 6014631.f1.pdf]
